# Early Onset of Type 1 Diabetes and Educational Field at Upper Secondary and University Level: Is Own Experience an Asset for a Health Care Career?

**DOI:** 10.3390/ijerph14070712

**Published:** 2017-06-30

**Authors:** Ida Lovén, Katarina Steen Carlsson

**Affiliations:** 1 Swedish Institute for Food and Agricultural Economics, Lund University, SE-220 07 Lund, Sweden; 2 Department of Clinical Sciences, Lund University, Malmö; Health Economics, SE-223 81 Lund, Sweden; Katarina.steen_carlsson@med.lu.se; 3 The Swedish Institute for Health Economics, SE-220 02 Lund, Sweden

**Keywords:** early life health, educational choices, comparative advantages, type 1 diabetes, long-term consequences, socioeconomic status, real world data, health data registers, Swedish Childhood Diabetes Study

## Abstract

Ill health in early life has a significant negative impact on school grades, grade repetition, educational level, and labor market outcomes. However, less is known about qualitative socio-economic consequences of a health shock in childhood or adolescence. We investigate the relationship between onset of type 1 diabetes up to age 15 and the probability of choosing and completing a health-oriented path at upper secondary and university level of education. We analyze the Swedish Childhood Diabetes Register, the National Educational Register, and other population registers in Sweden for 2756 people with type 1 diabetes and 10,020 matched population controls. Educational decisions are modeled as unsorted series of binary choices to assess the choice of educational field as a potential mechanism linking early life health to adult outcomes. The analyses reject the hypothesis of no systematic differences in choice of educational field between people with and without type 1 diabetes at both levels. The results are robust to selection on ability proxies and across sensitivity analysis. We conclude that the observed pro health-oriented educational choices among people with type 1 diabetes in our data are consistent with disease onset in childhood and adolescence having qualitative impact on life-course choices.

## 1. Introduction

Earlier work has shown that ill health in early life has a significant negative impact on school grades, grade repetition, educational level, and labor market outcomes (see, e.g., [1,2]). However, are all aspects of a health shock in childhood or adolescence necessarily bad, or could it also create comparative advantages and experiences that could have a professional value? Childhood and adolescence are formative years and the choices made could have life-long consequences [3]. The choice of educational path may be influenced by numerous factors within or beyond individuals’ (or their families’) control. Previous research connects cognitive abilities and socioeconomic background to both educational achievements (see, e.g., [1,4]) and labor market outcomes (see, e.g., [4,5]). Besides cognitive abilities, life experiences and preferences are likely drivers of choice. For example, Heckman, et al. [6] conclude that such non-cognitive abilities were equally important as cognitive abilities for an individual’s choice of schooling and wage (given the level of education chosen).

Traditionally, the economic literature has explored human capital in the form of years of schooling or attainment of a degree to assess its impact on life-time earnings (see, e.g., [5]). Complementary to formal education, abilities and skills created from life experiences could give the individual comparative advantages for specific careers [7]. Such comparative advantages could be decisive for paths of formal education and the choice of profession [8,9,10,11].

Drawing on the definition by Cunha and Heckman [3] of human capabilities as health, cognitive abilities, and non-cognitive abilities, we use onset of type 1 diabetes as a measure of change in human capabilities, to assess consequences for individuals’ choice of educational field. Health events early in life, such as the onset of chronic disease, are not in all aspects detrimental, but may also provide useful experiences and new capabilities. Disease onset can be expected to influence people in many dimensions. Yet, the impact of such experiences on educational choices has received little attention. On balance, a health shock early in life may reduce overall incentives for educational investments [2,12,13], but it could also create incentives for choosing an educational field where the experience of disease and its treatment could be an asset. Children often follow in their parents’ footsteps and it is long known that children of doctors often become doctors themselves [14,15]. This intergenerational transmission of career choices, as well as socioeconomic status more generally, occurs in part because preferences and information are transferred from parent to child [16]. Likewise, a child may acquire preferences and information through their own experiences of a chronic disease. Following this line of argument, own experience of disease would create a comparative advantage in health and medical professions from a qualitative perspective.

We contribute to the literature on child and adolescent health and adult outcomes by investigating whether experience of chronic disease influences educational choices. We analyzed the relationship between onset of type 1 diabetes (up to age 15) and the probability of choosing (and completing) a health-oriented educational program at upper secondary school and university. By modeling the educational decisions as an unsorted series of binary choices, we shed light on qualitative aspects of schooling and assess a potential mechanism linking early life health to adult outcomes. That is, we assess a potential change in human capabilities that was brought on by the onset of type 1 diabetes. Therefore, the interplay between an individual’s abilities and health are likely to be important for the studied health-education relationship, as confounders and/or mediators.

Salm and Schunk [17], Currie, Stabile, Manivong and Roos [13], and Heckman [18] argue that child or adolescent abilities are influenced by health and therefore have the potential to mediate the impact of health. Other researchers state that abilities may confound child health and adult outcomes as estimated effects are reduced when controlling for cognitive and non-cognitive ability [19,20]. Confounding seems reasonable for many health conditions driven by individuals’ behaviors and lifestyle. However, individuals are unable to influence or anticipate type 1 diabetes onset beforehand [21] and type 1 diabetes has therefore been described as an exogenous health shock [22,23,24]. Type 1 diabetes is triggered by a complex combination of genetic and environmental components that the individual needs to be exposed to in a certain sequence and during a vulnerable time period [21,25,26,27,28,29]. More than 90% of cases occur among individuals without a first-degree relative with the disease [30]. Empirical work indicate that the earnings penalty from diabetes is robust to sibling fixed effects and unobserved factors at the family level [31]. The shock-like nature of onset of type 1 diabetes supports the argument that abilities will mediate, rather than confound, the studied health-education relationship.

We investigate whether a health shock early in life links with a health-oriented education chosen at age 16 years or after age 18 years by comparing educational choices of people with type 1 diabetes to population controls with the same year of birth and municipality of residence in the year of disease onset. Our results reject the hypothesis of no systematic differences in choice of educational field between people with and without type 1 diabetes. The results are robust to selection on ability proxies and across sensitivity analysis.

## 2. Materials and Methods

### 2.1. Conceptual Framework

In contrast to the traditional human capital literature [32,33,34], which distinguishes between acquired skills and genetically determined cognitive ability, Cunha and Heckman [3], Heckman [18], and Cunha, Heckman, Lochner and Masterov [4] argue that behaviors and abilities have both a genetic and an acquired character. Measured abilities are the outcome of investments and gene- environment interactions. Abilities are cognitive (e.g., intelligence) or non-cognitive (e.g., patience, motivation, time preferences, and self-control) and they affect learning, health behaviors, and health. Thus, the human capabilities (health, cognitive abilities and non-cognitive abilities) are closely related and are formed throughout the life course. The model of human capability formation implies that, first, abilities are both persistent and self-reinforcing as higher abilities in one period lead to higher abilities in the next period and, second, previously acquired abilities make further investments more productive. Consequently, even small childhood health shocks might snowball into adverse adult health and labor market outcomes. Following, Cunha and Heckman [3], Heckman [18], and Cunha, Heckman, Lochner and Masterov [4], experiencing the onset of a chronic disease and new requirements of daily disease management could translate into a shift in preferences and comparative advantages for a health-oriented course of education.

### 2.2. Data

The Swedish Childhood Diabetes Register (SCDR) contains incident cases of type 1 diabetes in children aged 0–14.9 years in Sweden since 1 July 1977 [35] to enable studies on the etiology, incidence trends, and complications of diabetes. Data for the SCDR are collected according to the Declaration of Helsinki. To study socioeconomic effects of the onset of type 1 diabetes in childhood and adolescence, the Swedish Childhood Diabetes Study Group has added data to the SCDR as follows: for each individual in the SCDR, Statistics Sweden matched four people without diabetes from the Total Population Register by year of birth and municipality of residence at the time of type 1 diabetes diagnosis. Statistics Sweden identified parents of persons with type 1 diabetes and population controls from the Multi-Generation Register [36]. Covering the period 1990–2010, Statistics Sweden then added socioeconomic and demographic data for each person in the research database from the national population registers, the Longitudinal Integration Database for Health Insurance and Labour Market Studies (LISA) [37] and the Swedish Register of Education [38]. The merging of data and research was approved by the Regional Research Ethics Board in Umeå (Dnr 07-169M). All data provided to the research group have been anonymized by Statistics Sweden.

We retrieved data on 2756 individuals born in 1962–1975 and diagnosed with type 1 diabetes (hereafter referred to as diabetes) in the age group 2–15 (during the years 1977–1990) and 11,020 matched population controls.

#### 2.2.1. Dependent Variables

Onset occurs before entering upper secondary education as students enroll at age 16 [39]. We use the Swedish standard classification of education (SUN) [40], which is adapted to the International Standard Classification of Education 1997 (ISCED 97) [41], to identify level of education and aggregate programs into the different fields. We use the last available SUN registration up to year 2010. Appendix A lists the different fields available in SUN.

For university educations, we define two dichotomous dependent variables: *university attendance* that indicates if an individual have credits from a Swedish university corresponding to at least 20 weeks of full-time studies; and *health-oriented education* indicates university programs to become a physician, physiotherapist, occupational therapist, pharmacist, biomedical scientist, dentist, social worker, etc.

For upper secondary educations, the dependent upper secondary variable is an unordered categorical variable with four categories: *vocational health*, *vocational other*, *theoretical health*, and *theoretical other*. Vocational health includes programs that train for jobs in the health care sector, such as nursing assistant, but also jobs within social services, childcare and care for the elderly. Possibly, an interest in caring for other people, rather than an interest in health per se, has motivated some people to choose such programs, and we test the robustness of the results to the chosen classification in a sensitivity analysis. SUN registrations of upper secondary educations are not available in the data but we use the Swedish Register of Education and classify programs according to the SUN-classification. Theoretical programs at the upper secondary level prepare for all types of university studies and we use SUN-information on health orientation from subsequent choices of university education as a marker for health-oriented educations in the upper-secondary classification. For example, to become a physician, upper secondary studies in the general science program are required.

#### 2.2.2. Control Variables

We conduct analyses stratified by gender in line with the labor market literature and control for year of birth in three categories (1962–1965, 1966–1970, and 1971–1975) to capture (1) cohort competition in access to educational programs and jobs; and (2) economic trends and changes in the educational system. We also control for being born in a non-Nordic country and parents with non-Nordic origins, due to increased incidence of diabetes in the Nordic countries [42] and findings of labor market discrimination (see, e.g., Altonji and Blank [43]).

Child or adolescent abilities are influenced by health and therefore potentially mediate the impact of health [13,17,18]. If this is the case, the diabetes estimates will capture changes in individual characteristics due to the disease when abilities are not controlled for. We therefore test the robustness to observable background factors by assessing changes in the diabetes estimates when adding variables to our specification. The added background factors are likely to proxy ability as they correlate with socioeconomic status, health, and educational choices. Specification 1 controls only for year of birth and non-Nordic-origin and Specification 2 use the full set of controls.

The background factors include mother’s and father’s level of education (compulsory, upper secondary, and university) because parents contribute to their child’s health, abilities, and schooling [2,3,4,16,18,44]. Better-educated parents, as a group, have been found to have higher (acquired and/or innate) ability, earn more income, and live in areas providing high quality schooling. For the university-level analysis, we add a control for upper secondary grades, which may be a stronger ability proxy than parental education, but the grades are likely to be affected by the onset of diabetes. Moreover, parents’ choice of occupation might influence the child’s choice and we control for parents having a health-related upper secondary or university education (using the same classification as for the dependent variables).

Mother’s year of birth captures the increasing trend in women’s labor market participation since 1960 in Sweden, contributing to an increasing number of women working after they have had children [45]. Mother’s age at child’s birth takes into account that (1) younger mothers are less likely than older mothers to have had time to educate and establish themselves on the labor market; and (2) late childbearing increases the pregnancy-related risks for both mother and child [46,47]. Furthermore, women with low cognitive ability are more likely to bear children at younger ages [4].

### 2.3. Upper Secondary Education

We used a multinomial logit model to determine whether diabetes links to the probability of choosing (and completing) a health-oriented upper secondary education. In this setting, a child *i* gains utility from choosing an educational field *j* given his or her individual characteristics *x*:

(1) $$U_{ij}=\beta^{\prime }x_{i}+\;\varepsilon_{ij}$$

The child chooses the field that he or she prefers to all others and the probability that the child will choose field *j* is:

(2) $$Prob\left(Y_{i}=j\right)=\frac{\exp\left({\beta_{j}}^{\prime }x_{i}\right)}{\sum_{k=1}^{4}\exp\left({\beta_{k}}^{\prime }x_{i}\right)},\;j=1,\cdots ,4.$$

where the choice set *j* is: 1 = vocational other, 2 = vocational health, 3 = theoretical health, and 4 = theoretical other. Multinomial logit models were estimated using maximum likelihood to find the $\hat{\beta_{j}}$: s that best fit the data. We conditioned this analysis on having an upper secondary education. Consequently, each student falls into one of the educational fields and the probabilities will sum to one. To ensure model identification, *β*_1_ is set to zero (the reference category) and coefficients were interpreted with respect to vocational other (the largest group). The choice of reference category is important as the estimated coefficients apply in reference to that group. We found no indication that diabetes causes selection into upper secondary education or into vocational other. We tested for selection by estimating impacts of diabetes (dummy) on the likelihood of having (1) upper secondary education and (2) vocational other.

The advantage of the multinomial logit over other multinomial models is that the computations are simple and parameter estimates are relatively easy to interpret with marginal effects. The drawback is the restrictiveness of the independence of irrelevant alternatives (IIA) assumption. This assumption implies that the choice between any two pairs of alternatives is not affected if we add another alternative to the choice set or change the characteristics of a third alternative. It seems plausible to assume that, if someone has an interest in health, this interest remains even if the supply of educational programs in other fields changes. Still, individuals might have more than one career choice and, if the alternative choice becomes more accessible, one might favor this field instead. However, the IIA assumption is likely to hold when the categories of the dependent variables are sufficiently different. Our categories appear to be so, as a likelihood-ratio test and Wald test for combining outcome categories rejected the hypothesis that our independent variable did not differentiate between categories. Also, the two paths to a health-oriented education (vocational or theoretical) are likely to differ. A health-related occupation requiring a university education is arguably not the same as one requiring only upper secondary qualifications. To be accepted to a university after a vocational upper secondary program generally required supplementary studies regardless of field of education [48]. Nevertheless, to alleviate the IIA concerns, we tested the IIA assumption with a suest-based Hausman test and found no indication that the assumption is violated. Additionally, we ran a nested logit model that relaxed the IIA assumption. We grouped theoretical and vocational health into one nest and vocational and theoretical other into a second nest: thereby, we allowed the errors to be correlated within each nest, while assuming them to be independent between nests. We found similar results as when using the multinomial logit. Results are available in Appendix B and Appendix C.

### 2.4. University Education

For the university-level analysis, we modeled diabetes-related differences in (1) the probability of having a university education, and (2) the probability of having a health-oriented education for those with a university-level education. We used the logit model for these analyses as the outcomes are limited to choices between two alternatives.

### 2.5. Sensitivity Analyses

Two alternative definitions of health-oriented education explored the robustness of results. Lower grades and previous achievements might hinder individuals with diabetes from getting into popular educational programs. In addition, lower grades and attainment could be interpreted in terms of less productive learners or producers of abilities and deter those with diabetes from the more demanding programs. We cannot fully answer to the mechanisms at play, but we make a first attempt when testing the robustness of our results to (1) a more narrow definition of health-oriented education with a strict focus on health care and (2) the longest and most demanding university programs.

Further, we explored the impact of splitting the diabetes group by age at onset of disease. Evidence shows that children’s vulnerability to health shocks differs by age [1,4]. Duration of a disease might also be important, given that it generally takes time to adapt to new life circumstances [49] and for difficulties to manifest as, for example, lower educational achievements [23]. Crudely testing the results sensitivity to age of onset and consequently the duration disease when choosing educational path, we present results from analyses where the diabetes variable indicates onset at ages 2–9 or 10–15 (controls are still the reference). While individuals with onset in ages 10–15 were born in 1962–1975, individuals with onset in ages 2–9 were born during the years 1968–1975 due to the design of the data. Therefore, all individuals born before 1968 are excluded from this analysis.

## 3. Results

Table 1 shows the distribution of education. Comparing individuals with and without diabetes, there are no significant differences in upper secondary attendance, but a lower proportion of students with diabetes attended university (women and men *p* < 0.01). Women with diabetes are overrepresented in health-oriented education at all levels (*p* < 0.01), while men with diabetes are over-represented for vocational health but underrepresented for theoretical health (*p* < 0.01).

The descriptive statistics of the background factors in Table 2 show a higher proportion of non-Nordic-born individuals in the control groups than in the diabetes groups. Notably, there was no significant difference in parental level of education for women except for a tendency that mothers of women with diabetes have health-oriented education (*p* = 0.096). Parental education differed for men (mothers *p* = 0.014, fathers *p* = 0.002) as a consequence of the control group having a higher proportion of missing information on level of education, which was more common among people with a non-Nordic origin. Mothers in the diabetes group were on average older (women *p* = 0.076, men *p* = 0.068) and had children at older ages (women *p* = 0.0056, men *p* = 0.070) than mothers of controls. We tested if these differences imply confounding by regressing the independent variables on the probability of being in the diabetes group, but only non-Nordic-origin predicted diabetes (AME between −0.071 (*p* < 0.01) and −0.038 (*p* < 0.10) for parents, and −0.141 (*p* < 0.01) for own non-Nordic origin for men).

### 3.1. Upper Secondary Education

Table 3 for women and Table 4 for men show significant average marginal effects (AME) of diabetes on the probability of having upper secondary education in both vocational and theoretical health. The estimated AMEs are the change measured in percentage points. For instance, the estimated AME of diabetes on vocational health relative to vocational other was 0.055 (*p* < 0.01) which was then equivalent to a 5.5 percentage points increase in women’s likelihood of vocational health. The AME of diabetes on theoretical health was less strong (0.013; *p* < 0.10). Diabetes was associated with an increase in men’s likelihood of vocational health (0.031; *p* < 0.01), but the association is negative for theoretical health (−0.007; *p* < 0.01). Still, if we dichotomize the dependent variable into *health* and *non-health*, the results indicated that men have a general pro-health interest outweighing the lower interest for theoretical health as the AME of diabetes was positive for men (0.025; *p* < 0.01), as well as for women (0.067; *p* < 0.01).

Returning to Table 3 and Table 4, diabetes estimates were stable across the two model specifications, suggesting robustness to the influence of e.g., parental education and maternal ability. Parental level of education had the expected signs for both women and men. Mother’s year of birth and mother’s age at child’s birth appeared influential for women’s likelihood of having a health-oriented education, when simultaneously controlling for parental level of education. To have a mother with more recent year of birth was associated with an increasing likelihood of theoretical health, while the likelihood decreased for vocational health. Thus, women’s increased labor market participation appears to have a positive net effect on theoretical health, but a negative one for vocational health. Mother’s age at child’s birth is negatively associated with vocational health, suggesting that the likelihood of vocational health decreases with increasing age of the mother at the time of the child’s birth.

In summary, diabetes increased the likelihood of vocational health for both women and men, while the likelihood of theoretical health increased for women, but decreased for men. Both women (−0.063) and men (−0.039) with diabetes had a lower likelihood of theoretical other.

### 3.2. University Education

Table 5 and Table 6 present AME from logit estimations of the probability of having a university education (columns 1–2) and the probability of having a health-oriented education for those with a university education (columns 3–4). Without controlling for grades, diabetes was associated with a decreased likelihood of attending university (−0.064 for women, and −0.057 for men). When attending, the likelihood of health orientation increased for women (0.074) but not for men (the estimate was close to zero and insignificant). When adding controls for grades (column 2 vs. 1), the diabetes estimates on attendance were reduced by approximately one third: the AME of diabetes changed from −0.064 to −0.045 for women and from −0.057 to −0.044 for men. The diabetes estimates on the probability of having a health-oriented education were robust to the influence of grades. Higher grades were positively associated with attendance, but its link to educational field was weaker (partly because the analysis for health-orientation was conditioned on attendance, and grades likely affected university attendance).

Mothers with a health-oriented education along with parents’ level of education had larger estimates for attendance than for educational field, indicating that they were more linked to attendance. In contrast, having a father with a health-oriented education was related to health orientation (for men at least), while it appeared unrelated to attendance. Possibly, the differing influence of mothers’ and fathers’ health interest could relate to women and men generally having different types of occupations within the health care sector [50]. The influence of parents appeared less important once we add controls for grades, as both grades and parental level of education are proxies for abilities and skills.

### 3.3. Sensitivity Analyses

A more narrow definition of upper secondary vocational health education decreased the proportion of students from 15.7% to 7.4% but differences were negligible for theoretical health (decrease from 3.11% to 3.05%). The message of the main analysis remained, even though the shift substantially changed the distribution of educational fields: 47% of the diabetes group and 55% of the control group of main analysis vocational health switched category. Compared to the broader definition in Table 3 and Table 4, the estimates in Table 7 are smaller in size but remain significant for vocational health (for both women and men), while theoretical health becomes insignificant. 

Men and women with diabetes had a lower likelihood of attending university (Table 5 and Table 6). If this is a sign of lower levels of skills and abilities among the diabetes population, we would expect an even larger diabetes impact when analyzing the at the longest and most demanding university courses, such as the medicine program to become a physician, which demand top grades from upper secondary school science program. However, the results in Table 8 and Table 9 indicate that diabetes neither deterred nor excluded students from longer programs to greater extent than shorter programs. On the contrary, the negative diabetes estimates of the likelihood of attending university programs that are four years or longer were smaller in magnitude compared to the broader definition indicating that difference can be more prominent at intermediate rather that at the higher level of university education.

Among those attending for four or more years, there was no significant difference in health orientation, although the estimate for women maintained its size, and the power to detect differences was lower as the samples were smaller. Looking descriptively at average representation between women with and without diabetes on medicine programs, of those women who have a university education of four years or longer, 7.8% of the women with diabetes, compared to 7.7% of the controls, studied to become doctors, although these differences were not significant.

Table 10 and Table 11 present results from analyses where the diabetes-onset variable was split into two subcategories: onset at ages 2–9 years and 10–15 years (definition of control unchanged). For upper secondary education (Table 10), the diabetes estimates indicated a stronger association for onset in age 10–15 years than for onset at ages 2–9 years, although men with early-age onset appeared to be the ones driving the results for theoretical other. For university education (Table 11), the results for the two age at onset categories were mixed. Overall, these analyses support our main findings, as both early and late onset appear linked to upper secondary and university outcomes.

## 4. Discussion

We rejected the hypothesis of no systematic differences in the choice of educational field between people with diabetes onset up to age 15 and population controls. The pro-health orientation was found at both levels of education for women and at the upper secondary level for men. These findings are consistent with disease onset early in life having a qualitative impact on life-course choices.

The educational patterns of women and men in our control group (see Table 1) mirror the general population patterns for these birth cohorts presented by Statistics Sweden. For instance, national data for people aged 25–54 in 2010 showed that, at the upper secondary level, women are five times more likely than men to take a health-oriented examination and this over-representation is threefold for university degrees [51]. We found that women’s general pro-health orientation, was further accentuated among women with diabetes. To what extent this depended on stronger preferences for a health-oriented education, or on comparative advantages in achieving academic degrees, or both, was beyond the scope for this register-based research.

We note that the positive association for vocational health was larger for women than for men (AME 0.055, Table 3 compared to 0.031, Table 4). Moreover, there is a negative association for theoretical other that seems stronger for women (AME −0.063, Table 3 compared to −0.039, Table 4). A negative association for theoretical programs other than those related to health might relate to different interests, or the comparative advantages for a health-oriented education might have come to dominate over the other theoretical programs.

We used choice of educational field and completed health-oriented education as the dependent variable. In terms of returns to education and expected monthly salary from a future job, health-oriented programs prepare for a whole range of occupations: from low-wage occupations (e.g., nurse assistants and dental assistants), through middle-wage (e.g., physiotherapists and dental hygienist), to high-wage (e.g., physicians and dentists). Consequently, preferences for a future job in the health sector could satisfy a wide range of ambitions in terms of future labor earnings.

We used a clearly defined and well-described health shock, which requires subsequent daily lifelong disease management, as an indicator of health to assess the potential impact of ill health on preferences and comparative advantages for educational fields. The burden of disease management may trigger an interest for health-related professions, or deter from such professions as one might wish to separate one’s private and professional life. Our empirical results were consistent with both tendencies, and we found heterogeneous response to disease onset among women and men. The high coverage of the SCDR [52], together with universal health care at low cost (free in the case of pediatric care) and insulin free of charge to patients in Sweden ensures high representativeness, which is a strength compared to data from surveys and non-mandatory insurance data. Moreover, the health shock is assessed by a physician, and the impact on daily life and the health-related consequences are well-described [49,53], leaving no room for potential confusion with type 2 diabetes, which differs in etiology and key disease consequences.

Using data matched by municipality of residence in the year of onset and controlling for parental education and measures of maternal ability, we accounted, in part, for unmeasured attributes of the family and the environment during upbringing. Comparing specifications with and without these controls indicated a robust relationship between diabetes and choice of educational field. Our diabetes estimates decreased by about one third when we added upper secondary grades to the university attendance analysis, indicating that the diabetes impact was mediated partly by grades from upper secondary school, i.e., a post-onset indicator of ability.

In line with the literature on intergenerational transmissions of human capital [2,3,4,44,54], our results indicate that parental level of education was positively linked to the child’s own education (see Table 3, Table 4, Table 5 and Table 6), with an interesting pattern across the upper secondary fields. To have a university-educated mother was associated with a decreased likelihood of vocational health, but an increased likelihood of theoretical health and theoretical other. The opposite pattern was observed for mothers who had compulsory education: the likelihood decreased for theoretical health and theoretical other but increased for vocational health. Similar tendencies, but weaker in magnitude exist also for fathers as shown before by [4]. We are not aware of previous studies on parents’ differing influence for different educational fields. To have a mother with a health-oriented education appears to influence the child’s choice in a similar manner to having a mother with only compulsory education (the likelihood of vocational health was increased, but theoretical health was decreased).

Our choice of health indicator is one of the study’s strengths, while it could be argued that inference cannot be made with regard to other less well-defined health shocks with less clear impact on day-to-day activities. Consequently, these findings may not be representative for people with less demanding diseases, such as moderate asthma and allergy. However, milder disease or lower demands on daily management are arguably of less health policy concern regarding potential impact on educational and labor market outcomes.

The results of this study contribute to literature, including but not limited to earlier work from our group and from other Swedish based studies [23,55] and labor market outcomes [23,31,56,57], by analyzing more qualitative and preference based aspects of consequences of disease onset. We contribute to these studies on diabetes, as well as to the more general literature on early life health and adult outcomes (described in, e.g., [2]), by offering an alternative perspective. This study views experiences of disease not only as burden but also as a potential asset driving comparative advantages; thus, differing choices of educational field might explain why controlling for quantitative measures of schooling have had little impact on health effects in many previous studies [31,58].

This study introduces an alternative outcome where ranking in terms of desirability might not be obvious. For example, our results show that people with diabetes were less likely to have a university education. However, women with a university education were on average 7 percentage points more likely to have a health-oriented degree if they had diabetes. Own experience of disease and its treatment may accordingly translate into valuable assets and insights in working life. Still, the gender-related differences indicate a heterogeneous patient group.

## 5. Conclusions

Our findings support the argument that disease onset early in life may generate experiences and comparative advantages for choosing and completing a health-oriented course of education, both at upper secondary and university level. The results were robust in sub-group analysis and remained when controlling for school grades at the previous level.

## Figures and Tables

**Table 1:** Descriptive statistics of educational field at upper secondary school and university.

	Women			Men		
	Diabetes	Control		Diabetes	Control	
	*N* = 1292	*N* = 5444		*N* = 1464	*N* = 5576	
	Propor-tion	Propor-tion	*p*-Value ^1^	Propor-tion	Proport-tion	*p*-Value ^1^
*Upper secondary*						
Attendance	0.895	0.907	0.099	0.881	0.882	0.732
Type if attending			0.000			0.000
Vocational health	0.302	0.248		0.083	0.052	
Vocational other	0.318	0.321		0.613	0.597	
Theoretical health	0.061	0.048		0.006	0.013	
Theoretical other	0.318	0.383		0.298	0.338	
*University*						
Attendance	0.401	0.458	0.000	0.300	0.350	0.000
Type if attending			0.001			0.856
Health	0.303	0.233		0.075	0.073	
Other	0.697	0.767		0.925	0.927	

^1^ Attendance is tested with *t*-tests. Type if attending is tested with chi^2^ tests.

**Table 2:** Descriptive statistics of own and parents’ background factors. Numbers are proportions unless otherwise stated.

Variables	Women			Men		
	Diabetes *N* =1292	Control *N* = 5444	*p*-Value ^1^	Diabetes *N* = 1464	Control *N* = 5576	*p*-Value ^1^
Year of birth			0.040			0.067
Born 1962–65	0.056	0.076		0.090	0.072	
Born 1966–70	0.336	0.329		0.337	0.344	
Born 1971–75	0.608	0.595		0.573	0.584	
Non-Nordic	0.009	0.029	0.000	0.003	0.024	0.000
*Mothers*						
Level of education			0.100			0.014
Compulsory	0.321	0.335		0.351	0.344	
Upper secondary	0.444	0.429		0.426	0.424	
University	0.209	0.202		0.206	0.203	
Education missing	0.026	0.033		0.017	0.029	
Type of education						
Health	0.243	0.221	0.096	0.227	0.215	0.317
Non-Nordic	0.022	0.048	0.000	0.020	0.050	0.000
Year of birth (mean)	1944	1945	0.076	1944	1944	0.068
Age at child’s birth (mean)	27	26	0.006	27	26	0.070
*Fathers*						
Level of education			0.126			0.002
Compulsory	0.379	0.359		0.372	0.363	
Upper secondary	0.373	0.380		0.393	0.377	
University	0.186	0.186		0.185	0.184	
Education missing	0.062	0.076		0.051	0.075	
*Type of education*						
Health	0.026	0.026	0.942	0.025	0.022	0.489
Non-Nordic	0.056	0.083	0.001	0.055	0.087	0.000

^1^ Categorical variables is tested with chi^2^ test, others with *t*-test.

**Table 3:** Average marginal effects of women’s probability of having different fields of upper secondary education.

	Specification 1, *N* = 5952				Specification 2, *N* = 5846			
	Vocational Health	Vocational Other	Theoretical Health	Theoretical Other	Vocational Health	Vocational Other	Theoretical Health	Theoretical Other
Diabetes	0.055 ***	−0.0058	0.013 *	−0.063 ***	0.053 ***	−0.0032	0.014 *	−0.064 ***
	(0.015)	(0.015)	(0.0078)	(0.015)	(0.015)	(0.015)	(0.0078)	(0.015)
Ref. Control								
*Year of birth*								
1962–1965	0.080 ***	0.052 **	−0.020 **	−0.11 ***	−0.099 ***	0.018	0.0064	0.074
	(0.024)	(0.024)	(0.0099)	(0.024)	(0.037)	(0.044)	(0.024)	(0.049)
1966–1970	−0.0087	0.13 ***	−0.010 *	−0.11 ***	−0.097 ***	0.11 ***	0.0036	−0.015
	(0.012)	0.052 **	(0.0060)	(0.013)	(0.022)	(0.026)	(0.012)	(0.025)
Ref. 1971–1975								
*Mothers*								
Non-Nordic	−0.093 ***	−0.022	0.015	0.10 **	−0.10 ***	−0.014	0.020	0.095 **
	(0.033)	(0.039)	(0.020)	(0.043)	(0.034)	(0.041)	(0.021)	(0.043)
Ref. Nordic								
Year of birth					−0.018 ***	−0.00083	0.0022	0.017 ***
					(0.0042)	(0.0044)	(0.0021)	(0.0045)
Age at child’s birth					−0.019 ***	−0.0058	0.0029	0.022 ***
					(0.0043)	(0.0046)	(0.0022)	(0.0047)
*Level of education*								
Compulsory					0.024	0.058 ***	−0.025 ***	−0.057 ***
					(0.015)	(0.016)	(0.0061)	(0.015)
Ref. Upper secondary								
University					−0.072 ***	0.058 ***	0.033 ***	0.11 ***
					(0.015)	(0.016)	(0.0095)	(0.018)
Education missing					−0.047	0.058 ***	−0.052 ***	−0.0078
					(0.13)	(0.016)	(0.0045)	(0.13)
*Type of education*								
Health					0.083 ***	−0.0073	−0.010 *	−0.065 ***
					(0.016)	(0.016)	(0.0062)	(0.015)
Ref. Other								
*Fathers*								
Non-Nordic	−0.045 *	−0.013	0.025 *	0.033	−0.034	−0.022	0.025 *	0.031
	(0.024)	(0.026)	(0.015)	(0.027)	(0.025)	(0.026)	(0.015)	(0.027)
*Level of education*								
Compulsory					0.042 ***	0.020	−0.0047	−0.057 ***
					(0.014)	(0.014)	(0.0068)	(0.015)
Ref. Upper secondary								
University					−0.072 ***	−0.067 ***	0.022 ***	0.12 ***
					(0.016)	(0.017)	(0.0086)	(0.019)
Education missing					−0.031	0.063 **	0.0047	−0.037
					(0.024)	(0.028)	(0.013)	(0.027)
*Type of education*								
Health					0.026	−0.019	0.022	−0.029
					(0.040)	(0.041)	(0.017)	(0.036)
Ref. Other								

Robust standard errors in parentheses. *** *p* < 0.01, ** *p* < 0.05, * *p* < 0.1. Non-Nordic predicts failure perfectly and 171 observations are dropped.

**Table 4:** Average marginal effects of men’s probability of having different fields of upper secondary education.

	Specification 1, *N* = 6100				Specification 2, *N* = 5997			
	Vocational Health	Vocational Other	Theoretical Health	Theoretical Other	Vocational Health	Vocational Other	Theoretical Health	Theoretical Other
Diabetes	0.031 ***	0.015	−0.0070 **	−0.039 ***	0.032 ***	0.016	−0.0072 **	−0.041 ***
	(0.0083)	(0.015)	(0.0028)	(0.014)	(0.0084)	(0.014)	(0.0028)	(0.014)
Ref. Control								
*Year of birth*								
1962–1965	0.046 ***	−0.016	−0.011 ***	−0.019	0.031	−0.050	−0.0085	0.027
	(0.015)	(0.025)	(0.0030)	(0.024)	(0.031)	(0.047)	(0.0056)	(0.047)
1966–1970	−0.0070	0.15***	−0.0025	−0.14 ***	−0.013	0.12 ***	0.0019	−0.11 ***
	(0.0062)	(0.013)	(0.0030)	(0.012)	(0.012)	(0.024)	(0.0063)	(0.023)
Ref. 1971–1975								
*Mothers*								
Non-Nordic	−0.013	−0.044	0.0029	0.055	−0.014	−0.054	0.0069	0.060
	(0.017)	(0.041)	(0.0082)	(0.040)	(0.017)	(0.038)	(0.0099)	(0.038)
Ref. Nordic								
Year of birth					−0.0013	0.00079	0.00050	0.000016
					(0.0023)	(0.0044)	(0.0010)	(0.0042)
Age at child’s birth					−0.0015	−0.0047	0.00052	0.0057
					(0.0023)	(0.0045)	(0.0011)	(0.0043)
Level of education								
Compulsory					0.0071	0.086 ***	−0.0061 **	−0.087 ***
					(0.0081)	(0.015)	(0.0027)	(0.014)
Ref. Upper secondary								
University					0.0044	−0.15 ***	0.012 **	0.13 ***
					(0.0083)	(0.017)	(0.0047)	(0.017)
Education missing					0.034	−0.039	−0.011 ***	0.015
					(0.087)	(0.14)	(0.0021)	(0.13)
*Type of education*								
Health					0.018 **	−0.15 ***	0.0016	−0.074 ***
					(0.0085)	(0.017)	(0.0032)	(0.013)
Ref. Other								
*Fathers*								
Non-Nordic	0.015	−0.025	0.011	−0.0011	0.017	−0.027	0.011	−0.0018
	(0.015)	(0.027)	(0.0079)	(0.025)	(0.015)	(0.026)	(0.0079)	(0.025)
Ref. Nordic								
Level of education								
Compulsory					−0.0068	0.10 ***	0.0012	−0.099 ***
					(0.0073)	(0.014)	(0.0032)	(0.014)
Ref. Upper secondary								
University					0.0020	−0.18 ***	0.012 ***	0.17 ***
					(0.0094)	(0.019)	(0.0043)	(0.018)
Education missing					−0.0036	0.084 ***	0.0061	−0.086 ***
					(0.014)	(0.027)	(0.0068)	(0.025)
Type of education								
Health					0.0014	0.034	0.020 *	−0.055 *
					(0.019)	(0.037)	(0.011)	(0.033)
Ref. Other								

Robust standard errors in parentheses. *** *p* < 0.01, ** *p* < 0.05, * *p* < 0.1. Year of birth in 1971–1975 and Upper secondary education are reference categories. Non-Nordic predicts failure perfectly and 140 observations are dropped.

**Table 5:** Average marginal effects of women’s probability of having a university education and the probability of having a health-related university education for those with a university education.

	University, *N* = 6508		Health Related, *N* = 2946	
	(1)	(2)	(3)	(4)
Diabetes	−0.064 ***	−0.045 ***	0.074 ***	0.076 ***
	(0.015)	(0.014)	(0.022)	(0.022)
Ref. Control				
Grades from upper secondary level		0.26 ***		0.034 **
		(0.0091)		(0.015)
Grades missing		0.37 ***		0.22 ***
		(0.010)		(0.076)
Ref. Grades non-missing				
*Mothers*				
Non-Nordic	0.0071	0.030	0.037	0.035
	(0.036)	(0.034)	(0.050)	(0.049)
Ref: Nordic				
Year of birth	0.0013	−0.0016	−0.0096	−0.0097
	(0.0043)	(0.0040)	(0.0059)	(0.0059)
Age at child’s birth	0.0096 **	0.0023	−0.011 *	−0.012 *
	(0.0044)	(0.0041)	(0.0061)	(0.0061)
*Level of education*				
Compulsory	−0.14 ***	−0.094 ***	−0.012	−0.011
	(0.015)	(0.014)	(0.022)	(0.022)
Ref. Upper secondary school				
University	0.18 ***	0.12 ***	−0.032	−0.037 *
	(0.017)	(0.016)	(0.019)	(0.019)
Education missing	−0.13	0.0029	−0.12	−0.13
	(0.088)	(0.089)	(0.13)	(0.12)
*Type of education*				
Health	−0.061 ***	−0.031 **	0.018	0.020
	(0.014)	(0.013)	(0.020)	(0.020)
Ref. Other				
*Fathers*				
Non-Nordic	0.014	0.026	−0.013	−0.016
	(0.026)	(0.023)	(0.033)	(0.033)
Ref. Nordic				
*Level of education*				
Compulsory	−0.052 ***	−0.031 **	0.047 **	0.046 **
	(0.014)	(0.013)	(0.020)	(0.020)
Ref. Upper secondary				
University	0.14 ***	0.089 ***	−0.0060	−0.0080
	(0.018)	(0.017)	(0.020)	(0.020)
Education missing	−0.082 ***	−0.032	0.022	0.014
	(0.025)	(0.023)	(0.038)	(0.038)
*Type of education*				
Health	−0.0099	0.0089	0.078	0.077
	(0.037)	(0.036)	(0.048)	(0.048)
Ref. Other				
Demographics	Yes	Yes	Yes	Yes

Robust standard errors in parentheses. *** *p* < 0.01, ** *p* < 0.05, * *p* < 0.1. Demographics—year of birth, non-Nordic.

**Table 6:** Average marginal effects of men’s probability of having a university education and the probability of having a health-related university education for those with a university education.

	University *N* = 6854		Health-Related *N* = 2313	
	(1)	(2)	(3)	(4)
Diabetes	−0.057 ***	−0.044 ***	0.0046	0.0049
	(0.013)	(0.012)	(0.015)	(0.015)
Ref. Control				
Grades from upper secondary level		0.20 ***		0.012
		(0.0079)		(0.0100)
Grades missing		0.34 ***		0.044
		(0.021)		(0.061)
Ref. Grades non-missing				
*Mothers*				
Non-Nordic	0.0087	−0.0014	0.044	0.042
	(0.033)	(0.031)	(0.040)	(0.039)
Ref. Nordic				
Year of birth	0.00093	−0.0021	0.0012	0.0011
	(0.0039)	(0.0037)	(0.0040)	(0.0040)
Age at child’s birth	0.0095 **	0.0035	−0.00091	−0.0011
	(0.0041)	(0.0038)	(0.0040)	(0.0040)
*Level of education*				
Compulsory	−0.11 ***	−0.086 ***	−0.0040	−0.0041
	(0.013)	(0.013)	(0.014)	(0.014)
Ref. Upper secondary				
University	0.15 ***	0.096 ***	0.0046	0.0027
	(0.017)	(0.015)	(0.014)	(0.014)
Education missing	0.010	0.095		
	(0.099)	(0.093)		
*Type of education*				
Health	−0.051 ***	−0.035 ***	0.011	0.012
	(0.013)	(0.012)	(0.014)	(0.014)
*Fathers*				
Non-Nordic	−0.0027	0.0039	0.058 **	0.057 *
	(0.024)	(0.022)	(0.029)	(0.029)
*Level of education*				
Compulsory	−0.099 ***	−0.076 ***	0.052 ***	0.052 ***
	(0.013)	(0.012)	(0.017)	(0.017)
University	0.17 ***	0.13 ***	0.00074	−0.00072
	(0.018)	(0.016)	(0.013)	(0.013)
Education missing	−0.10 ***	−0.053 **	0.023	0.022
	(0.022)	(0.022)	(0.026)	(0.026)
*Type of education*				
Health	−0.0070	−0.019	0.083 *	0.082 *
	(0.031)	(0.030)	(0.044)	(0.044)
Ref. Other				
Demographics	Yes	Yes	Yes	Yes

Robust standard errors in parentheses. *** *p* < 0.01, ** *p* < 0.05, * *p* < 0.1. Demographics—year of birth, non-Nordic.

**Table 7:** Test with a more restrictive definition of health-oriented education: Average marginal effects of the probability of having different fields of upper secondary education.

	Women, *N* = 5952				Men, *N* = 6100			
	Voca-tional Health	Voca-tional Other	Theore-tical Health	Theore-tical Other	Voca-tional Health	Voca-tional Other	Theore-tical Health	Theore-tical Other
Diabetes	0.049 ***	−0.00013	0.010	−0.059 ***	0.021 ***	0.025 *	−0.0067 **	−0.040 ***
	(0.012)	(0.016)	(0.0075)	(0.015)	(0.0051)	(0.015)	(0.0028)	(0.014)
Ref. Control								
*Year of birth*								
1962–1965	0.011	0.12 ***	−0.027 ***	−0.11 ***	0.0053	0.025	−0.014 ***	−0.017
	(0.018)	(0.026)	(0.0091)	(0.024)	(0.0061)	(0.024)	(0.0019)	(0.024)
1966–1970	0.016 *	0.10 ***	−0.012 **	−0.11 ***	0.0084 **	0.13 ***	−0.0025	−0.14 ***
	(0.0097)	(0.014)	(0.0060)	(0.013)	(0.0035)	(0.013)	(0.0030)	(0.012)
Ref. 1971–1975								
*Mothers*								
Non-Nordic	−0.026	−0.088 **	0.0087	0.11 **	0.0052	−0.063	0.0029	0.055
	(0.028)	(0.041)	(0.019)	(0.043)	(0.012)	(0.040)	(0.0081)	(0.040)
Ref. Nordic								
*Fathers*								
Non-Nordic	−0.021	−0.037	0.027 *	0.031	0.0014	−0.012	0.011	−0.00095
	(0.018)	(0.028)	(0.015)	(0.027)	(0.0072)	(0.026)	(0.0078)	(0.025)
Ref. Nordic								

Robust standard errors in parentheses. *** *p* < 0.01, ** *p* < 0.05, * *p* < 0.1. Non-Nordic predicts failure perfectly and 311 observations were dropped.

**Table 8:** Average marginal effects of women’s probability of having a university education longer than four years and the probability of having a health-related university education longer than four years for those with a university education longer than four years.

	University 4+ Years, *N* = 6508		Health Related 4+ Years, *N* = 730	
	(1)	(2)	(3)	(4)
Diabetes	−0.027 ***	−0.019 **	0.044	0.045
	(0.0090)	(0.0089)	(0.043)	(0.043)
Ref. Control				
Grades from upper secondary level		0.13 ***		0.019
		(0.0066)		(0.029)
Grades missing		0.58 ***		0.066
		(0.025)		(0.14)
*Year of birth*				
1962–1965	0.020	0.0059	−0.13	−0.14
	(0.033)	(0.029)	(0.084)	(0.084)
1966–1970	0.0056	−0.00075	−0.062	−0.063
	(0.017)	(0.016)	(0.058)	(0.058)
Ref. 1971–1975				
Non-Nordic	0.027	0.038	0.024	0.031
	(0.039)	(0.038)	(0.12)	(0.12)
Ref. Nordic				
*Background factors*				
Mother	Yes	Yes	Yes	Yes
Father	Yes	Yes	Yes	Yes

Robust standard errors in parentheses. *** *p* < 0.01, ** *p* < 0.05. Background factors includes the full set of controls for mothers and fathers.

**Table 9:** Average marginal effects of men’s probability of having a university education longer than four years and the probability of having a health-related university education longer than four years for those with a university education longer than four years.

	University 4+ Years, *N* = 6858		Health Related 4+ Years, *N* = 654	
	(1)	(2)	(3)	(4)
Diabetes	−0.024 ***	−0.016 **	0.0025	0.0066
	(0.0078)	(0.0075)	(0.028)	(0.029)
Ref. Control				
Grades from upper secondary level grades		0.13 ***		0.036
		(0.0055)		(0.023)
Grades missing		0.58 ***		0.50
		(0.028)		(0.31)
*Year of birth*				
1962–1965	−0.0050	−0.012	−0.072 *	−0.072 *
	(0.025)	(0.023)	(0.039)	(0.039)
1966–1970	−0.0039	−0.00039	−0.014	−0.016
	(0.014)	(0.013)	(0.046)	(0.045)
Ref. 1971–1975				
Non-Nordic	−0.066 ***	−0.050 *		
	(0.020)	(0.029)		
Ref. Nordic				
*Background factors*				
Mother	Yes	Yes	Yes	Yes
Father	Yes	Yes	Yes	Yes

Robust standard errors in parentheses. *** *p* < 0.01, ** *p* < 0.05, * *p* < 0.1. * Non-Nordic predicts failure perfectly and 3 observations are dropped. Background factors include the full set of controls for mothers and fathers.

**Table 10:** Test with two diabetes onset categories: Average marginal effects of the probability of having different fields of upper secondary education.

	Women, *N* = 4897				Men, *N* = 4993			
	Vocational Health	Vocational Other	Theoretical Health	Theoretical Other	Vocational Health	Vocational Other	Theoretical Health	Theoretical Other
Diabetes								
Onset 2–9	0.050 **	−0.028	0.020	−0.042	0.026 *	0.033	−0.0034	−0.056 **
	(0.025)	(0.025)	(0.014)	(0.026)	(0.014)	(0.026)	(0.0060)	(0.025)
Onset 10–15	0.074 ***	0.015	0.0036	−0.093 ***	0.030 ***	0.021	−0.0089 **	−0.042 **
	(0.020)	(0.020)	(0.0099)	(0.020)	(0.011)	(0.020)	(0.0035)	(0.019)
Ref. Control								
*Year of birth*								
1968–1970	−0.029 **	0.15 ***	−0.016 **	−0.11 ***	−0.013 **	0.16***	−0.00077	−0.15 ***
	(0.014)	(0.015)	(0.0066)	(0.015)	(0.0067)	(0.015)	(0.0036)	(0.014)
Ref. 1971–1975								
*Mothers*								
Non-Nordic	−0.093 ***	0.015	−0.0047	0.082 *	−0.021	−0.063	−0.00068	0.084 *
	(0.036)	(0.045)	(0.018)	(0.047)	(0.016)	(0.043)	(0.0081)	(0.043)
Ref Nordic								
*Fathers*								
Non-Nordic	−0.046 *	−0.029	0.035 **	0.040	0.0055	−0.017	0.014	−0.0025
	(0.025)	(0.027)	(0.017)	(0.030)	(0.014)	(0.029)	(0.0095)	(0.028)
Ref. Nordic								

Robust standard errors in parentheses. *** *p* < 0.01, ** *p* < 0.05, * *p* < 0.1. Non-Nordic predicts failure perfectly and 311 observations are dropped.

**Table 11:** Test with two diabetes onset categories: Average marginal effects of the probability of having a university education (Uni) and the probability of having undertaken a health-related university program for those with a university education (Health).

	Women		Men	
	University *N* = 5368	Health at University *N* = 2477	University *N* = 5609	Health at University ^1^ *N* = 1952
Diabetes				
Onset 2–9	−0.034	0.095 **	−0.069 ***	−0.031
	(0.025)	(0.037)	(0.023)	(0.021)
Onset 10–15	−0.089 ***	0.074 **	−0.042 **	0.021
	(0.019)	(0.030)	(0.017)	(0.021)
Ref. Control				
*Year of birth*				
1968–1970	0.0016	−0.024	0.00066	0.0063
	(0.025)	(0.033)	(0.023)	(0.024)
Ref. 1971–1975				
Non-Nordic	−0.045	−0.075	−0.074	
	(0.057)	(0.070)	(0.057)	
Ref. Nordic				
*Background factors*				
Mother	Yes	Yes	Yes	Yes
Father	Yes	Yes	Yes	Yes

Robust standard errors in parentheses. *** *p* < 0.01, ** *p* < 0.05. Background factors include the full set of controls for mothers and fathers. Non-Nordic predicts failure perfectly and 34 observations are dropped.

## Appendix A

### The SUN-classification system

List of educational programs in the SUN-classification system:

1. Universal educational programs at upper secondary level preparing for higher education, including natural science, and humanistic and social science
2. Pedagogy and pedagogical work
3. Art and culture
4. Social science, law, commerce, administration
5. Natural science, mathematics, computers and network technology
6. Engineering and manufacturing
7. Natural resources and forestry
8. Health, medical, and social care
9. Services
10. Other

### Vocational health includes programs from item 8 (health, medical, and social care).

(1) Vocational health
  (a) health and medicine
  (b) dental care
  (c) child care
  (d) social work
  (e) care and treatment
(2) Vocational health restrictive
  (a) health and medicine
  (b) dental care

*Theoretical health* includes programs from item 1 (universal educational programs) if they are followed by a university-level program from item 8 (health, medical, and social care).

## Appendix B

### Tests of the IIA assumption

Ho: Odds (Outcome-J vs. Outcome-K) are independent of other alternatives.

**Table A1:** Suest-based Hausman tests of the IIA assumption.

Omitted	chi^2^	df	*p* > chi^2^	Evidence
*Result for women*				
Voc. health	5.363	12	0.945	for Ho
Theo. health	15.256	12	0.228	for Ho
Theo. other	6.701	12	0.877	for Ho
*Result for men*				
Omitted	chi^2^	df	*p* > chi^2^	evidence
Voc. health	3.864	12	0.986	for Ho
Theo. health	5.834	12	0.924	for Ho
Theo. other	5.766	12	0.927	for Ho

## Appendix C

### Nested logit models

**Table A2:** Average marginal effects (AME) by nested logit estimation of the probability of field of upper secondary education using vocational other as the reference category. Estimations control for year of birth and own and parental non-Nordic-origin.

Outcome	Diabetes AME	Std. Dev.
*Result for women*		
Vocational health	0.064	0.016
Theoretical health	0.005	0.004
Theoretical other	−0.061	0.007
*Result for men*		
Vocational health	0.031	0.006
Theoretical health	−0.007	0.002
Theoretical other	−0.039	0.007
